# Intubation in prone position using AirTraq Avant videolaryngoscope

**DOI:** 10.1007/s10877-018-0128-1

**Published:** 2018-03-19

**Authors:** Tomasz Gaszynski

**Affiliations:** Department of Anaesthesiology and Intensive Therapy, Medical University of Lodz, Lodz, Poland

To the Editor,

One of the life-threatening situations during surgery is unintentional tracheal extubation of patient under general anaesthesia in position limiting face access and can cause serious complications if not followed by rapid reintubation [1]. Sudden extubation which may happen in patient in prone position for example during general anaesthesia for spine surgery may lead to serious complications. There is still no best method of management of these situation in clinical settings [1]. A securely fastened reinforced cuffed endotracheal tube is still considered the airway of choice for patients in the prone position [2]. Although some authors suggest that routine intubation using standard Macintosh blade laryngoscope in prone position is possible we think that it can be performed effectively and safely only by experienced anaesthesiologists, requires continuous training [3]. There are number of papers confirming successful use of supraglottic devices for airway management in anaesthesia patients in prone position. For exapmle Weksler et al. observed neither complications nor airway loss when LMA was used in the prone position [4]. In the interesting brief review of Abrishami et al. authors concluded that cumulative experience from published reports suggests the feasibility of placing the supraglottic device with the patient in the prone position in the elective setting; however, the evidence is lacking regarding the use of this method for emergency management of unintended tracheal extubation with the patient in the prone position [5]. Sudden extubation or malfunction of tracheal tube in anaesthetized patient in prone position usually the management included positioning patient in supine position and reintubation. However, this may lead to complications related to repositioning the patient with ongoing surgery and the complications related to possible hypoxia during positioning the patient from prone to supine position. Videolaryngoscopes present new promising possibilities.

**Fig. 1:**
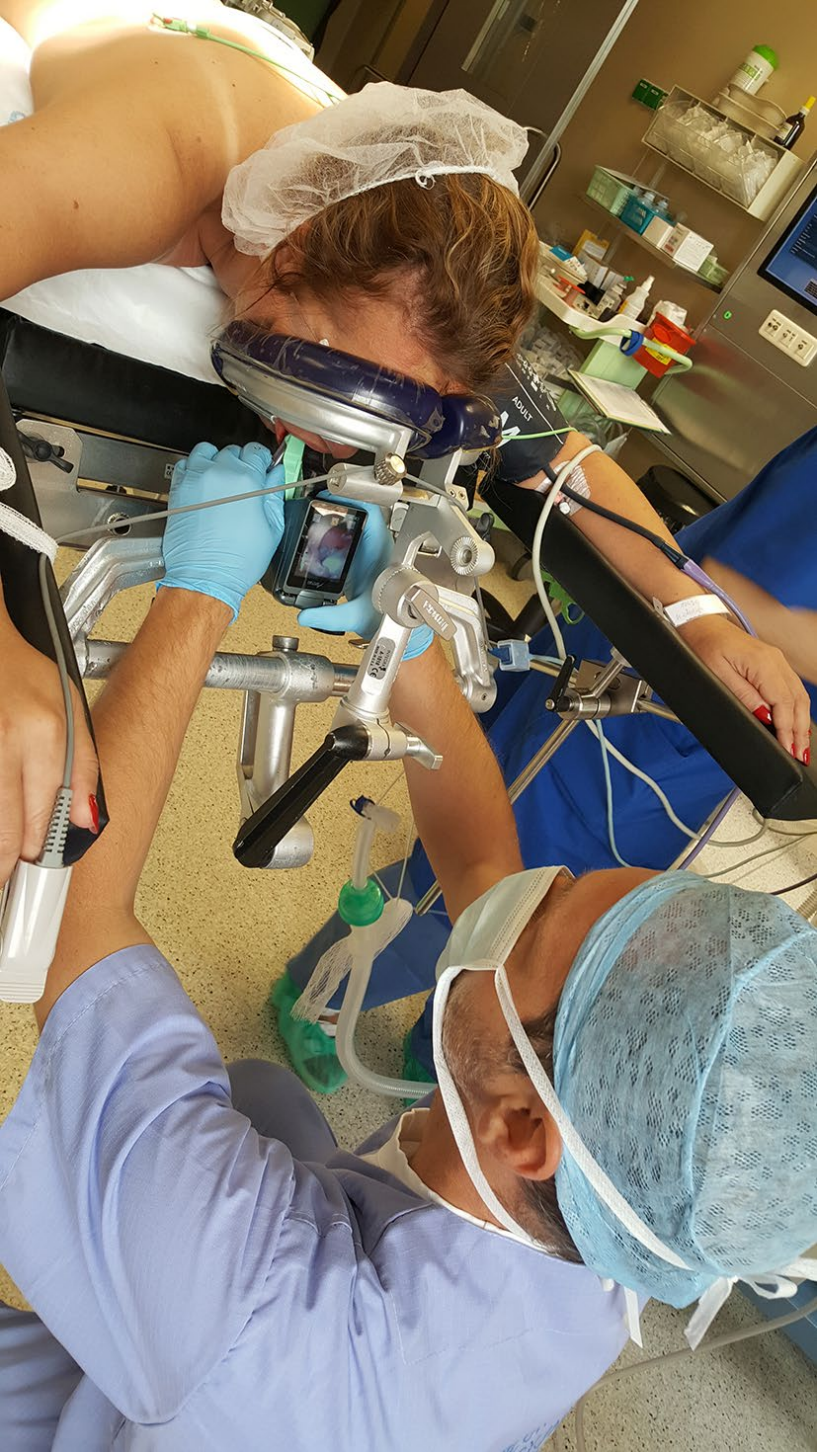
Intubation in prone position using AirTraq Avant videolaryngoscope

We would like to present the case of successful reintubation of patient in prone position using AirTraq Avant videolaryngoscope. In the 39 years female patient (170 cm, 74 kg) anaesthetized and intubated using standard Macintosh blade laryngoscope without any difficulties in supine position for elective lumbal spine surgery shortly after positioning in prone position for surgery a serious leak was detected in anaesthesia circuit due to malfunction of endotracheal tube cuff. We decided to attempt intubation in prone position using AirTraq Avant videolaryngoscope (Fig. 1). The intubation was fast and safe. There was no complications.

There is no other paper on using videolaryngoscopes for intubation of patients in prone position. Kaur et al. reported successful use of C-Mac videolaryngoscope for intubation of patient in lateral position [6]. Wang et al. used for intubation in lateral position Glidescope videolaryngoscope [7]. Linton et al. proposed in their algorithm of anaesthesia management of patients with pressure ulcers in prone position endotracheal intubation using videolaryngoscopes as more safe and effective [8]. Videolaryngoscopes proved to be useful in case of difficult situations like emergency reintubation [9], however, they differ in construction and therefore can provide different effectiveness of intubation [10]. In case of failed direct laryngoscopy as rescue device videolaryngoscopes with extra-curved blade are preferred [11]. We must admit that intubation using videolaryngoscope in patient in prone position requires skills in videolaryngoscopy. The operator should be familiar with videolaryngoscope is going to use. Potential difficulties that may face during intubation attempts in prone positioned patient are: lack of space around the mouth of the patient because of head holder and difficult access to patient`s head because of prone position. The possible solution of the problem may be introducing first blade and then connecting the videolaryngoscope as we did in presented case. Videolaryngoscope blade can be introduced similar way to introducing oropharyngeal tube. Secondly the operator must be aware that maneuvering with videolaryngoscope in this conditions (patient facing down) is reverse comparing to patient positioned in supine position. For example to elevate epiglottis it is necessary to pull down (not up) the blade. This may be confusing for not experienced operators. And lastly not every videolaryngoscope seams to fit for attempts of intubation patients in prone position for example because of space around patient`s face and neck needed to introduce device into mouth of the patient.

Channelled videolaryngoscopes may be more adequate in case of intubation patients in prone position, however further observation are needed.
